# N-Methyl-D-Aspartate (NMDA) Receptors in the Prelimbic Cortex Are Required for Short- and Long-Term Memory Formation in Trace Fear Conditioning

**DOI:** 10.3390/life12050672

**Published:** 2022-05-01

**Authors:** Eui-Ho Park, Nam-Soo Kim, Yeon-Kyung Lee, June-Seek Choi

**Affiliations:** 1 Department of Psychology, Korea University, Seoul 02841, Korea; euiho@korea.ac.kr (E.-H.P.); kimn2@janelia.hhmi.org (N.-S.K.); epleasure@korea.ac.kr (Y.-K.L.); 2 Department of Physiology, College of Medicine and Neuroscience Research Institute, Korea University, Seoul 02841, Korea; 3 Department of Psychology and Neuroscience, Duke University, Durham, NC 27708, USA

**Keywords:** prelimbic cortex, N-methyl-D-aspartate (NMDA) receptors, trace fear conditioning

## Abstract

Accumulating evidence suggests that the medial prefrontal cortex (mPFC) has been implicated in the acquisition of fear memory during trace fear conditioning in which a conditional stimulus (CS) is paired with an aversive unconditional stimulus (UCS) separated by a temporal gap (trace interval, TI). However, little is known about the role of the prefrontal cortex for short- and long-term trace fear memory formation. Thus, we investigated how the prelimbic (PL) subregion within mPFC in rats contributes to short- and long-term trace fear memory formation using electrolytic lesions and d,l,-2-amino-5-phosphonovaleric acid (APV), an N-methyl-D-aspartate receptor (NMDAR) antagonist infusions into PL. In experiment 1, pre-conditioning lesions of PL impaired freezing to the CS as well as TI during the acquisition and retrieval sessions, indicating that PL is critically involved in trace fear memory formation. In experiment 2, temporary blockade of NMDA receptors in PL impaired the acquisition, but not the expression of short- and long-term trace fear memory. In addition, the inactivation of NMDAR in PL had little effect on locomotor activity, pre-pulse inhibition (PPI), or shock sensitivity. Taken together, these results suggest that NMDA receptor-mediated neurotransmission in PL is required for the acquisition of trace fear memory.

## 1. Introduction

In typical Pavlovian fear conditioning, an initially neutral conditioned stimulus (CS) is contingently paired with an aversive unconditioned stimulus (US). As a result, a conditioned response (CR) emerges rapidly to the CS in a form of a defensive reaction such as freezing or autonomic activation, indicative of long-lasting plasticity for processing the relevance of the CS to the upcoming threat of the US. Key brain circuits including the thalamo-amygdala and hippocampo-amygdala pathways have been proposed to contribute to the formation and storage of associative fear memory [1,2,3]. 

A variation of this procedure, namely trace fear conditioning (TFC) where the CS and the US are paired with the temporal gap, has gained much attention as this procedure seems to require several brain areas for overcoming this temporal gap. In fact, much evidence has shown that not only the amygdala [4], but also the hippocampus [5,6,7,8,9,10], entorhinal cortex [11], and perirhinal cortex [12] can involve TFC. 

The medial prefrontal cortex (mPFC) has been suggested as a critical structure for the acquisition and consolidation of trace memory, mainly in eyeblink conditioning. Infusion of muscimol, a γ-amino-butyric acid type A (GABAA) agonist into mPFC, disrupted the acquisition of trace, but not delay eyeblink conditioning [13]. In addition, N-methyl-d-aspartate receptors (NMDARs)-dependent synaptic plasticity of mPFC is required for consolidation of trace memory, as the infusions of the NMDA receptor antagonist d-2-amino-5-phosphonovaleric acid (d-APV) into the mPFC immediately after trace eyeblink conditioning impaired conditioned response (CR) in the retention test [14]. Recent studies also reported that the inactivation of NMDARs in mPFC impaired the acquisition of conditioned trace, but not delay, fear responses [15]. Specifically, inactivation of NR2A- but not NR2B containing NMDARs interfered with the formation of trace fear memory [16]. 

Within mPFC, anatomically and functionally distinct subregions, namely the prelimbic (PL) and infralimbic (IL) areas have been identified as they send projections to different areas of the brain [17]. Regarding their role in fear conditioning, IL sends a heavy excitatory input to GABAergic cells in LA (lateral amygdala) and ITC (intercalated cell cluster) that exert inhibitory inputs to the central amygdala (CE). The regulation of learned fear response during extinction has been extensively investigated in relation to IL [17,18]. In contrast, PL sends excitatory projections into the basal amygdala (BA) which activates CE and importantly sends projections back to PL [19]. Interestingly, micro-stimulations of PL, contrary to IL, modulate fear response or impaired fear extinction [20]. Accordingly, PL has been implicated in context-dependent modulation of fear response [21].

Given the modulatory role of PL in the acquisition of fear response, we investigated whether NMDAR-dependent neurotransmission in the PL contributes to short-term or long-term trace fear memory formation. In the first experiment, we conducted the electrolytic lesion of PL before trace fear conditioning, to confirm that PL is required for the trace fear memory formation. In the second experiment, we tested whether NMDAR-dependent synaptic transmission is critical for either the short- or long-term trace fear memory formation or both by micro-infusions of APV into PL bilaterally.

## 2. Materials and Methods

### 2.1. Animals

Male Sprague-Dawley rats (250~300 g; *n* = 89; Orient Bio, Seongnam, Korea) were individually placed in a temperature- and light-controlled vivarium (22–25 °C, 12-h light/dark cycle) and were allowed *ad libitum* access to water and food. All experiments were conducted during the dark phase of the cycle and followed the ethical guidelines established by the Guide for the Care and Use of Laboratory Animals (http://oacu.od.nih.gov/regs/index.htm, accessed on 1 January 2019). All animal use procedures were conducted according to the guidelines of the Institutional Animal Care and Use Committee at Korea University (approval number: KOREA-2018-0060-C1 and KOREA-2018-0064-C1).

### 2.2. Surgery

For electrolytic lesion, each rat was anesthetized with sodium pentobarbital (60 mg/kg, i.p.) and mounted on a stereotaxic apparatus (David Kopf Instruments, Tujunga, CA, USA). The electrolytic lesions in the prelimbic (PL) (3.5 mm anterior, ±0.6 mm lateral, and 3.8 mm ventral to bregma) were made bilaterally for PL-lesion group (PL-lesion) bypassing anodal current (1.0 mA for 15 s) delivered through the stainless-steel electrode insulated with Epoxylite except for the tip (0.5 mm). In contrast, the sham group received the same insertion of the electrode as the PL-lesion except that no current was passed.

For cannula implantation, each rat was fully anesthetized with sodium pentobarbital (60 mg/kg, i.p.) and mounted on the stereotaxic apparatus. Bilateral guide cannulae (26 gauge, Plastics One, Roanoke, VA, USA) were implanted into the PL at coordinates 3.2 mm posterior, ±0.5 mm lateral, and 3.0 mm ventral to the bregma. The cannulae were anchored in place with dental acrylic. Dummy cannulae (33 gauge; Plastics One) were inserted 0.5 mm below the guide cannulae to prevent tissue entry. The rats were allowed to recover for at least 7 days following surgery before the experiments.

### 2.3. Drug Infusion

DL-2-amino-5-phosphnovaleric acid (APV, 10 μg/μL; Sigma, St. Louis, MO, USA) as the competitive NMDA receptor antagonist was dissolved in the artificial cerebrospinal fluid (aCSF) and injected bilaterally into the PL. After the tips of infusion cannulas (33 gauge; Plastics One, Roanoke, VA, USA) were gently placed 0.5 mm above the target coordinates through the implanted cannulas, the drug was infused at a rate of 0.25 μL/min for 2 min (0.5 μL per hemisphere) by a programmable infusion pump (model 101, KD Scientific, Hollistion, MA, USA). Infusion cannulas were left for the diffusion of APV for 3 min, and then removed from the implanted cannulas. The drug infusions were conducted 10 min before the tests. All procedures have been tested successfully in our previous study [22].

### 2.4. Conditioning Apparatus

Two different conditioning chambers (30 cm × 25 cm × 25 cm, four black Plexiglas walls; 27 cm × 25 cm × 34 cm, two transparent Plexiglas and two aluminum walls), each surrounded by sound-attenuating cabinets, were used. The grid floor of each chamber had 18 stainless steel rods (4 mm diameter, 1.5 cm apart) wired to a shock generator (E13–14, Coulbourn Instrument, Allentown, PA, USA). A speaker (8 cm × 4 cm, 8 Ω) mounted on the sidewall delivered the tone CS and a video camera on the ceiling recorded the behavior. Ventilation fans on the back wall of the cabinet provided background noise (60 ± 2 dB).

### 2.5. Behavioral Procedure and Analysis

**Figure 1:**
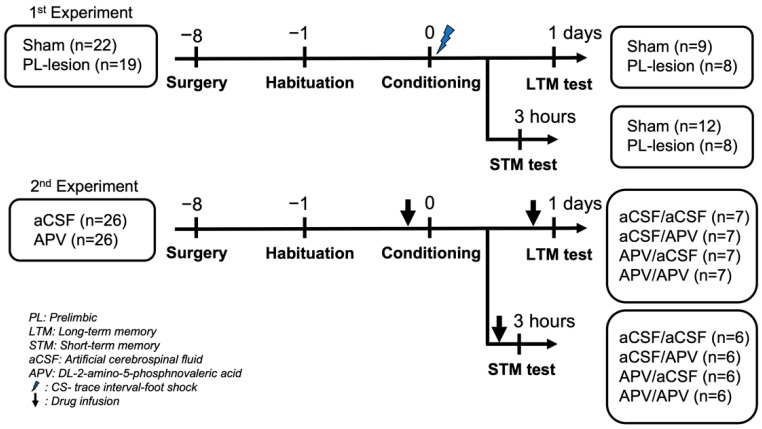
Experimental schedules for conditioning and surgery with electrolytic lesions (1st) and drug injection (2nd).

As shown in Figure 1, Sham (*n* = 22) and PL-lesion (*n* = 16) groups received the habituation, conditioning, and retention tests in experiment 1. On day 1, the rats were habituated to the conditioning chamber for 30 min. On day 2, the rats were given the baseline period (240 s) in the chamber after which they received seven pairings of the CS (2 kHz, 15 s, 75 ± 2 dB measured from inside the chamber) were paired with foot-shocks (US; 0.5 mA, 1 s) interposed by a stimulus-free period (TI, 30 s). The inter-trial interval (ITI) was pseudo-randomized within 3 min. After the conditioning, the rats were returned to their home cages.

For the retention test, the rats were placed in a test chamber in which contextual cues such as illuminations (red or blue light), floors (stainless steel rod or Plexiglas floor), and odorants (clean air, cinnamon, or resin) were changed to eliminate contextual freezing. The rat was given a baseline period of 240 s. After the baseline period, the seven tones without foot shock were delivered in the schedule of 3 min ITI. Some rats (sham, *n* = 12; PL-lesion, *n* = 8) were assigned for STM retention test (3 h after the conditioning) and others (sham, *n* = 9; PL-lesion, *n* = 8) were involved in LTM retention test (1 day after the conditioning). All experimental procedures used in the current study have produced reliable responses in previous studies from our laboratory [4,10].

In the second experiment, all procedures were the same as in the first experiment: habituation (day 1), conditioning (day 2), and retention tests (STM or LTM). After the habituation, the rats received injections of aCSF or DL-2-amino-5-phosphonovaleric acid (APV, 10 μg/μL; Sigma, St. Louis, MO, USA), a competitive NMDA receptor antagonist injection into the PL before conditioning or before retention tests (conditioning/STM: aCSF/aCSF, *n* = 7; aCSF/APV, *n* = 7; APV/aCSF, *n* = 7; APV/APV, *n* = 7; conditioning/LTM: aCSF/aCSF, *n* = 6; aCSF/APV, *n* = 6; APV/aCSF, *n* = 6; APV/APV, *n* = 6). Freezing, defined as the absence of movement except for respiration, was measured as the index of CR [23]. Freezing during the CS and the TI was recorded by a video recording, and then quantified by two experimenters who were blind to the condition of the subjects using digital stopwatches.

### 2.6. Open Field, PPI and Shock-Sensitivity Test

One week after trace fear conditioning, locomotor activity was assessed in a square arena (77 × 77 × 25 cm^3^). The center of the open field was defined as a square section in the middle of the arena (46.2 × 46.2 cm^2^). After aCSF (*n* = 7) or APV (*n* = 8) was bilaterally injected into mPFC 10 min before the test, the rat was allowed to explore freely the area for 10 min. A webcam monitored and recorded the animal movement. Moving distance and the proportion of occupation between central and marginal areas were analyzed using an automated tracking system (SmarTrack; Smartech, Madison, WI, USA).

For PPI (pre-pulse inhibition) test, aCSF (*n* = 7) or APV (*n* = 8) was bilaterally injected into the mPFC. At 10 min after the injection, the rat was restrained in acryl cylinder (10 cm in diameter, 20 cm in length). The load cell (CB1-K002, Dacell Co., Cheongju, Chungcheongbuk-Do, Korea) platform established beneath the restrainer in the sound-attenuated chamber measured the startle response. The test was comprised of 15 blocks of four different trial types with an average ITI of 15 s: the pre-pulse (75 dB) preceding the startle stimulus (120 dB) by 100 ms; pre-pulse alone; the startle stimulus alone; and no stimulus. PPI was calculated as follows: PPI (%) = (1 − [(response for startle stimulus with the pre-pulse)/(response for startle stimulus only)]) × 100.

For the shock-sensitivity test, such as open field and PPI tests, aCSF (*n* = 7) or APV (*n* = 8) was bilaterally injected into the mPFC 10 min before the test. Each rat received a series of foot shocks (1 s duration, 10 s ITI) in which the shock began at 0.1 mA and progressed by 0.05 mA increments until the rat exhibited reactions such as flinching, hopping, jumping, running, and sonically vocalizing.

### 2.7. Statistical Analysis

Freezing was normalized as a percentage of time during the presentation of CS and TI. For all experiments, behavioral differences were analyzed using ANOVA or Student’s *t* tests. In case of significant main effects, Tukey *post hoc* comparisons were conducted.

## 3. Results

### 3.1. Lesions and Drug Injection Sites

**Figure 2:**
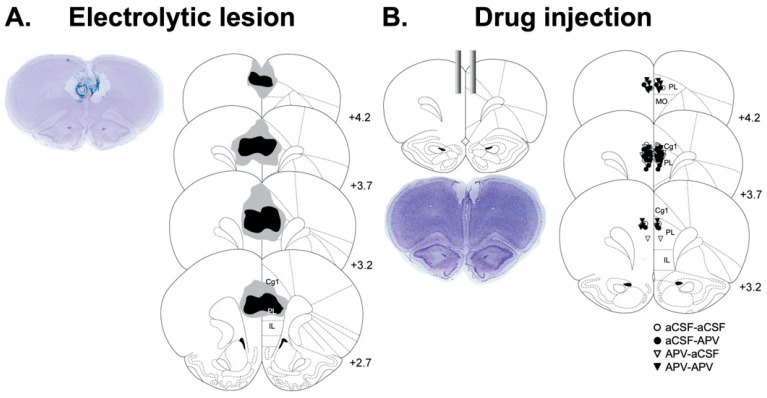
Electrolytic lesions and drug injection sites. (**A**) Reconstructions of the most extensive (gray) and least extensive (black) lesions in coronal section (bregma 2.7, 3.2, 3.7 and 4.2 mm) are illustrated. (**B**) Locations of cannula tip in coronal section (bregma 3.2, 3.7 and 3.70 mm) are marked in PL. Anterior extension of cingulate cortex area 1 (Cg1), prelimbic area (PL), infralimbic, medial orbitofrontal cortex (MO), artificial cerebrospinal fluid (aCSF), and DL-2-amino-5-phosphnovaleric acid (APV).

Figure 2 shows representative photomicrographs of coronal sections from electrolytic lesions in PL (Figure 2A) and cannula implantations for drug injection (aCSF/APV) (Figure 2B). A quantitative analysis using NIH ImageJ 1.41 revealed that the PL-lesion group was damaged on the anterior extension of cingulate cortex area 1(Cg1, 11.2 ± 1.8%), prelimbic area (PL, 51.5 ± 3.3%), infralimbic (IL, 1.4 ± 0.8%) and medial orbitofrontal cortex (MO, 3.4 ± 0.9%). The tips of injection cannulas were targeted within the PL.

### 3.2. PL Is Necessary for Short- and Long-Term Trace Fear Memory Formation

**Figure 3:**
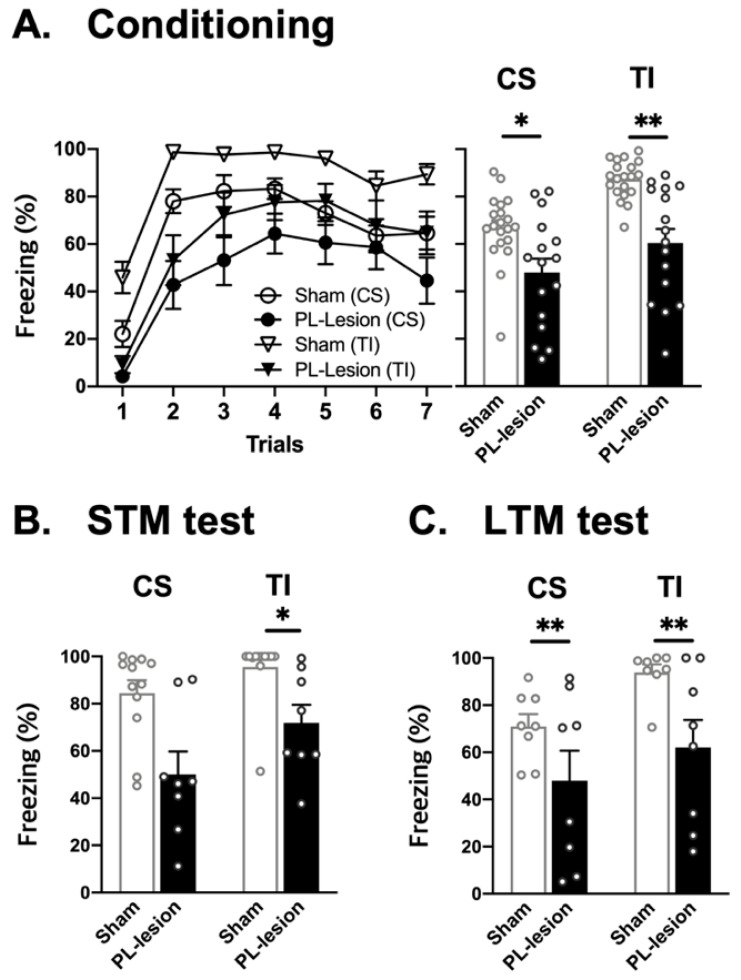
Effects of PL lesion on conditioning and retention tests. (**A**) Freezing response to the conditional stimulus (CS) and trace interval (TI) between sham and PL-lesion groups during conditioning session. (**B**) Freezing response to the CS and TI during STM test. (**C**) Freezing response to the CS and TI during LTM test. Values are expressed as mean ± SEM. * *p* < 0.05 and ** *p* < 0.01 vs. Sham group.

To test whether PL is required for the CS-UCS association over the trace interval (TI), the rats received either sham (sham: *n* = 22) or electrolytic lesions of PL (PL-lesion = 16) before the conditioning test. During the conditioning, a repeated ANOVA analysis revealed that there were significant group effect [CS, *F*(1, 34) = 8.575, *p* < 0.01; TI, *F*(1, 34) = 22.502, *p* < 0.01], and conditioning effect [CS, *F*(6, 204) = 22.08, *p* < 0.01; TI, *F*(6, 204) = 33.236, *p* < 0.01]. In addition, Student’s t test showed that PL-lesion group is significantly impaired in CS-UCS association over the TI compared to Sham group [CS, *t*(34) = 2.928, *p* < 0.05; TI, *t*(34) = 4.744, *p* < 0.01] (Figure 3A).

To test whether PL is required for either short- or long-term trace fear memory formation, some rats received the STM test (sham: *n* = 12, PL-lesion: *n* = 8), and others received LTM test (sham: *n* = 9, PL-lesion: *n* = 8). For the STM test, PL-lesion group showed significantly decreased freezing to the TI, but not to the CS, compared to sham group (TI, *t*(18) = 2.976, *p* < 0.05; Figure 3B). For LTM test, freezing behavior to the CS and the TI were significantly impaired in PL-lesion compared to sham group (CS, *t*(14) = 1.664, *p* < 0.01; TI, *t*(14) = 2.612, *p* < 0.01; Figure 3C). This result indicates that PL is critical for trace memory formation.

### 3.3. NMDA Receptors in PL Is Critical for Short- and Long-Term Trace Fear Memory Formation

**Figure 4:**
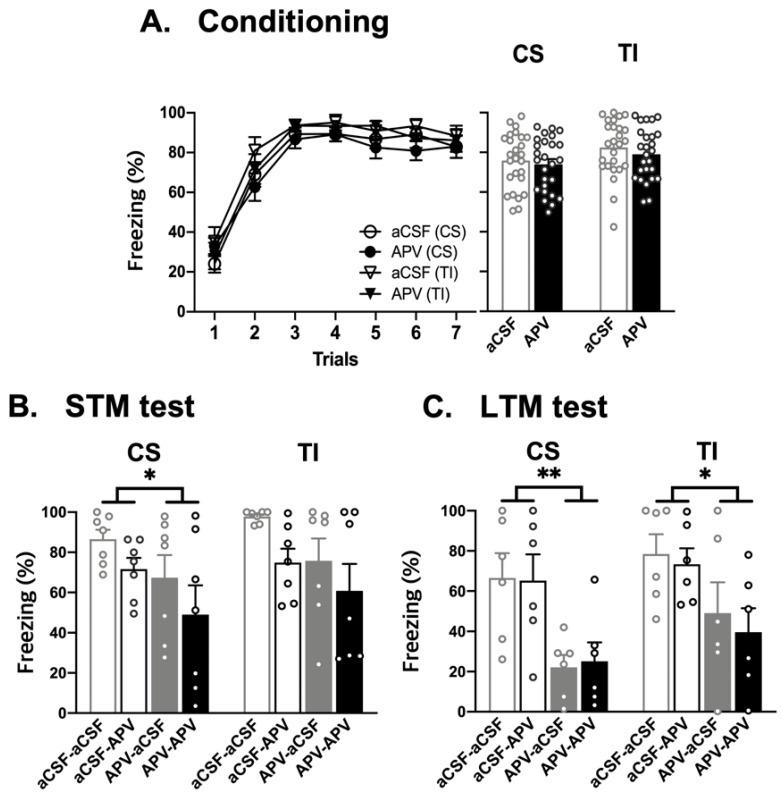
Effects of drug injection into PL on conditioning and retention tests. (**A**) Freezing response to the CS and TI between aCSF- and APV-treated groups before conditioning session. (**B**) Freezing response to the CS and TI between aCSF- and APV-treated groups before STM test. (**C**) Freezing response to the CS and TI between aCSF- and APV-treated groups before LTM test. Values are expressed as mean ± SEM. * *p* < 0.05 or ** *p* < 0.01 means a significant main effect of pre-conditioning drug infusion.

To address the contribution of NMDA receptors in PL to the association of CS and UCS over the temporal gap, APV or aCSF was injected into PL before the conditioning test. During the conditioning, both APV and aCSF groups showed increased freezing over trials. A repeated ANOVA analysis showed that there were significant conditioning effect [CS, *F*(6, 300) = 51.337, *p* < 0.01; TI, *F*(6, 300) = 45.576, *p* < 0.01], but no main effect of drug [CS, *F*(1, 50) = 0.225, *p* = 0.637; TI, *F*(1, 50) = 0.812, *p* = 0.372] or interaction [CS, *F*(1, 50) = 0.551, *p* = 0.462; TI, *F*(1, 50) = 0.217, *p* = 0.643] (Figure 4A). To confirm, Student’s *t* test compared the average level of freezing between the two groups for the CS and TI. There was no significant difference between APV and aCSF groups during the CS [CS, *t*(50) = 0.474, *p* = 0.637] or during the TI [*t*(50) = 0.901, *p* = 0.372] (Figure 4A). The result indicates that the blockade of NMDA receptors in the PL had no effect on freezing during the conditioning session, perhaps due to foot shock-induced freezing.

Following the conditioning, the rats were randomly divided into two groups: short-term memory (STM) and long-term memory (LTM) groups. Rats in the STM were further divided into two groups, receiving APV or aCSF infusion immediately before the STM test (*n* = 7 for all four groups). Rats in LTM received the same treatment except for the timing of drug infusion and the retrieval test (*n* = 6 for all four groups). For STM groups, a two-way ANOVA revealed that there was a significant main effect of pre-conditioning drug infusion on the level of freezing to the CS (*F*(1, 24) = 4.466, *p* < 0.05) but not to the TI. There was no significant main effect of pre-test drug infusion nor interaction (Figure 4B. STM test). For LTM groups, a two-way ANOVA revealed that there was a significant main effect of pre-conditioning drug infusion on the level of freezing to the CS (*F*(1, 20) = 15.783, *p* < 0.01) as well as to the TI (*F*(1, 20) = 7.842, *p* < 0.05). There was no significant main effect of pre-test drug infusion nor interaction (Figure 4C. LTM test). Post hoc analysis showed that APV/aCSF group had significantly reduced freezing to the CS than that to aCSF/aCSF (*p* < 0.05) and aCSF/APV *(p* < 0.05) for the LTM groups. None of the other comparisons were significant. To sum, pre-conditioning infusions of APV significantly reduced freezing to the CS and TI during STM and LTM tests regardless of the type of drug-infused before the retrieval test. These results indicate that the pre-conditioning effect of drug infusion is not due to state-dependent expression of freezing response.

### 3.4. The Blockade of NMDA Receptors for Behavioral States

To examine that the acquisition deficit of trace fear memory could be caused by the hyperactivity or the deficit of responsiveness to a tone or a foot-shock per se following APV injections, we performed open field, PPI, and shock sensitivity tests one week after trace fear conditioning. For the open field test, aCSF and APV groups showed no difference in the distance, marginal and central duration [distance, *t*(13) = −1.749, *p* = 0.104; marginal duration, *t*(13) = −0.425, *p* = 0.678; central duration, *t*(13) = 0.423, *p* = 0.679] (not shown). For PPI test, aCSF and APV groups showed no difference in PPI response [*t*(13) = 0.365, *p* = 0.721] (not shown). For the shock sensitivity test, the sensitivity to the foot-shock was categorized as flinching, hopping, jumping, running, and sonically vocalizing, which were assessed by minimal intensity required to express such unconditioned responses [24]. aCSF and APV groups showed no difference in behavioral responses to the foot-shock [flinch, *t*(13) = 0.001, *p* = 1.00; hop, *t*(13) = 0.327, *p* = 0.749; jump, *t*(13) = −0.851, *p* = 0.41; run, *t*(13) = 0.161, *p* = 0.874; sonic vocalization, *t*(13) = −1.238, *p* = 0.238] (not shown). These results support that the acquisition deficit of trace fear memory results from the blockade of NMDA receptors, but not hyperactivity or attention deficit.

## 4. Discussion

In the current study, we found that PL is required for short- and long-term trace fear memory formation. Furthermore, we found that NMDA receptors (NMDARs) in PL are critical for the acquisition and/or early consolidation, but not the expression, of short- and long-term trace fear memory formation. The role of NMDARs in PL in trace conditioning is confined to the memory formation as the blockade of NMDARs had little effect on locomotion, responses to the tone (PPI), or the shock (shock sensitivity).

Previous studies have suggested that neural plasticity in mPFC (IL/PL) is necessary for the retention or recall of extinction memory formation following fear conditioning [25]. For example, electrolytic lesions of mPFC resulted in retarded extinction of conditioned fear response to the CS [26]. In addition, preventing protein synthesis in mPFC or activating protein kinases modulated consolidation of extinction memory [27,28]. Moreover, the activities of IL neurons are distinctively activated during the retrieval of extinction memory [29] and high-frequency stimulation of mediodorsal thalamic inputs to mPFC maintained the extinction [30]. However, recent studies in rats targeted the role of mPFC on fear response; their neurons were activated in the acquisition of olfactory conditioning [29], and the activation of extracellular signal-regulated kinase (ERK) in the mPFC correlated with the storage of the trace fear memories [31].

NMDARs have been known for controlling synaptic plasticity and memory function [32]. Specifically, NMDARs in the hippocampus mediate fear memory association. To support, temporary inactivation of NMDARs in the dorsal hippocampus impaired contextual and trace fear memory. Knockout mice lacking NMDARs in the CA1 area of the hippocampus showed impaired conditioned responses in trace fear conditioning [33,34]. More pertinent to the current study, NMDARs in the mPFC critically contribute to trace fear memory formation [15], especially when the long-term fear memory was tested. In addition, intracerebroventricular application of the NMDAR antagonist, APV, before training interfered with fear conditioning when tested 24 h later but not during the conditioning session, indicating that the blockade of the NMDARs did not diminish shock-induced freezing [35,36]. Controversially, other studies have shown that NMDARs might be necessary for both short- and long-term memory. A study has shown that intraperitoneal application of MK-801 (NMDA antagonist) impaired both short- and long-term retention of object recognition memory when given pre- or post-training [37]. More interestingly, recent studies have shown that the defect in memory function induced by NMDAR antagonists resulted in the impairment in the acquisition of new information [38,39] or in the consolidation of short-term memory into long-term memory [2]. Moreover, consolidation of fear extinction also requires post-training activation of NMDAR in mPFC [40]. Furthermore, a study claimed that short-term memory test is sensitive to NMDA receptor antagonist type, dosage, and interval between acquisition and retention [41]. Thus, further investigation is needed to elucidate the contribution of the NMDA receptor on short-term trace fear memory.

In some views, impaired trace fear response induced by the inactivation of the NMDA receptors in the PL can be attributable to the problems with sensory processing per se or hyperactivity, but not with mnemonic formation. Alternatively, it might be suggested that PL lesions or NMDA receptors inactivation were extended into the anterior cingulate cortex (ACC) and thus impaired neuronal processes to focus the attention on auditory stimuli or receive nociceptive input through the spinothalamic pathway [42,43,44]. However, our results show that the sensitivity to an auditory tone (PPI) or foot shock (US) and basal locomotor activity was not influenced by the blockade of NMDARs in PL.

## Data Availability

Not applicable.
